# Birth outcomes, puberty onset, and obesity as long-term predictors of biological aging in young adulthood

**DOI:** 10.3389/fnut.2022.1100237

**Published:** 2023-01-10

**Authors:** Martin Jáni, Lenka Zacková, Pavel Piler, Lenka Andrýsková, Milan Brázdil, Klára Marečková

**Affiliations:** ^1^Brain and Mind Research, Central European Institute of Technology, Masaryk University, Brno, Czechia; ^2^Department of Psychiatry, Faculty of Medicine, Masaryk University and University Hospital Brno, Brno, Czechia; ^3^Department of Neurology, St. Anne’s University Hospital and Faculty of Medicine, Masaryk University, Brno, Czechia; ^4^RECETOX, Faculty of Science, Masaryk University, Brno, Czechia

**Keywords:** biological aging, BMI, obesity, puberty, birth outcomes, life history theory

## Abstract

**Background:**

Biological aging and particularly the deviations between biological and chronological age are better predictors of health than chronological age alone. However, the predictors of accelerated biological aging are not very well understood. The aim was to determine the role of birth outcomes, time of puberty onset, body mass index (BMI), and body fat in accelerated biological aging in the third decade of life.

**Methods:**

We have conducted a second follow-up of the Czech part of the European Longitudinal Study of Pregnancy and Childhood (ELSPAC-CZ) prenatal birth cohort in young adulthood (52% male; age 28–30; *n* = 262) to determine the role of birth outcomes, pubertal timing, BMI, and body fat on biological aging. Birth outcomes included birth weight, length, and gestational age at birth. Pubertal timing was determined by the presence of secondary sexual characteristics at the age of 11 and the age of first menarche in women. Biological age was estimated using the Klemera-Doubal Method (KDM), which applies 9-biomarker algorithm including forced expiratory volume in one second (FEV1), systolic blood pressure, glycated hemoglobin, total cholesterol, C-reactive protein, creatinine, urea nitrogen, albumin, and alkaline phosphatase. Accelerated/decelerated aging was determined as the difference between biological and chronological age (BioAGE).

**Results:**

The deviations between biological and chronological age in young adulthood ranged from −2.84 to 4.39 years. Accelerated biological aging was predicted by higher BMI [in both early (R^2^_*adj*_ = 0.05) and late 20s (R^2^_*adj*_ = 0.22)], subcutaneous (R^2^_*adj*_ = 0.21) and visceral fat (R^2^_*adj*_ = 0.25), puberty onset (η_*p*_^2^ = 0.07), birth length (R^2^_*adj*_ = 0.03), and the increase of BMI over the 5-year period between the two follow-ups in young adulthood (R^2^_*adj*_ = 0.09). Single hierarchical model revealed that shorter birth length, early puberty onset, and greater levels of visceral fat were the main predictors, together explaining 21% of variance in accelerated biological aging.

**Conclusion:**

Our findings provide comprehensive support of the Life History Theory, suggesting that early life adversity might trigger accelerated aging, which leads to earlier onset of puberty but decreasing fitness in adulthood, reflected by more visceral fat and higher BMI. Our findings also suggest that reduction of BMI in young adulthood slows down biological aging.

## 1. Introduction

We live in an era of unprecedented aging (1). The percentage of people aged 65 and higher worldwide was 9% in 2019 and is expected to rise to 12% by 2030 and to 16% by 2050 (2). This increase in lifespan brings a proportional increase in age-related disease (3, 4). Previous research suggested that age-related changes in the organism accumulate well before the onset of disease and that even early life factors contribute to the speed of aging (5–7). In order to intervene early, a better understanding of the age-related changes and early detection of the altered aging trajectory is crucial (8).

To measure the aging process, US National Health and Nutrition Survey (NHANES) studied participants aged 30–75 years and developed a 10-biomarker-based measure of “Biological Age”, which was more successful in predicting mortality in the 20-year follow-up than chronological age (9). Using the NHANES algorithm, Belsky et al. (8) calculated the Biological Age of Dunedin Study members and found variations in biological aging in young individuals of the same chronological age. While all participants were 38 years old, their biological age varied from 28 to 61 years (8). Higher biological vs. chronological age was associated with poorer physical fitness, appearance, and cognitive decline (8). The current study aims to find predictors of such accelerated biological aging.

Growing evidence in the last decade suggests that higher Body mass index (BMI) can have detrimental effect on life expectancy (10–12). Obesity has been linked to multiple chronic diseases, reduced functional capacity and lower quality of life (11–14). It is thus of no surprise that anti-aging strategies proposed to extend lifespan focus on caloric restriction (15). Promising results have been reported in primates, but their effectiveness is yet to be verified. However, shared epigenetic signatures (e.g., histone modification, DNA methylation, non-coding RNAs, and chromatin remodeling) have been reported in obesity and aging (16), suggesting BMI might be a possible predictor of biological aging.

Higher BMI in adulthood was associated with earlier onset of puberty (17, 18), another important predictor of all-cause and cardiovascular mortality (19, 20). According to Belsky (21, 22) early pubertal maturation and accelerated biological aging are part of the same evolutionary-developmental process, i.e., Life History Theory. Recent research supported this theory by demonstrating accelerated epigenetic aging in women with earlier onset of puberty (5, 23).

According to Belsky and Shalev (22, 24), earlier pubertal maturation is the result of faster biological aging that stems from adverse/stressful events early in life. Further research on aging and timing of puberty reported that child maltreatment (sexual, physical, or emotional abuse) predicts earlier onset of puberty in women (25) and is associated with accelerated epigenetic aging (5). Earlier pubertal maturation was also found in women who reported more risky and uncertain environments early in life (26) and in the offspring of mothers who reported depression symptoms, marital conflict, and financial stress during pregnancy (27). Consistently, an independent line of research associated higher mortality with preterm birth (28) and small body size indicated by small ponderal index (29), suggesting that birth outcomes might be among the key predictors of biological aging.

This emerging evidence suggests that higher mortality in adulthood is associated with accelerated biological aging, which might have its roots in early life. The current study aims to use data from the European Longitudinal Study of Pregnancy and Childhood (ELSPAC-CZ) prenatal birth cohort (30) and its two follow-ups in young adulthood [VULDE, Health Brain Age (7)] to determine the role of birth outcomes, time of puberty onset, BMI, and body fat in accelerated biological aging in the third decade of life. Since earlier pubertal development has been reported in women compared to men (31) and previous studies showed different trajectory of fat distribution between men and women during pubertal development (32) that continue with aging (33), we also considered potential sex differences in the relationships.

## 2. Materials and methods

### 2.1. Participants

A total of 262 young adults (52% men, 28–30 years of age; all of European ancestry) participated in the Health Brain Age project at the Central European Institute of Technology, Masaryk University (CEITEC MU), a follow-up of the Czech part of the European Longitudinal Study of Pregnancy and Childhood (ELSPAC-CZ) (30), a prenatal birth cohort born in the South Moravian Region of the Czechia between 1991 and 1992. A subset of these participants (*n* = 110, 51% men) also took part in the first follow-up of this prenatal birth cohort at the age of 23–24 years, entitled Biomarkers and Underlying Mechanisms of Vulnerability to Depression (VULDE; *n* = 131) (7), and thus have a within-subject design data regarding anthropometrics in young adulthood. A diagram illustrating the sample size of the different studies as well as the final sample of the current study is provided in Supplementary Figure 1. Men and women did not differ in any of the demographic variables; detailed characteristics of the Health Brain Age sample can be found in Table 1 and descriptive statistics and sample size included in the different analyses can be found in Table 2. All participants gave written informed consent for participation in Health Brain Age and VULDE (when applicable) and agreed to merge their historic data from ELSPAC-CZ and the subsequent studies. Informed consent was approved by the ELSPAC Ethics Committee.

**TABLE 1:** Demographic information.

Demographics	Men	Women	Between group differences
	(*N* = 136)	(*N* = 126)	
**Ethnicity**			
White Caucasian	100%	100%	n/a
**Age**			
in years	M = 28.96 (±0.67)	M = 28.99 (±0.69)	t(260) = 0.34, *p* = 0.732
**Education**			
Not completed high school	2%	2%	χ^2^(3) = 5.94, *p* = 0.110
Completed high school	29%	18%	
Completed university	66%	79%	
Completed postgradual education	2%	1%	
Missing	0%	0%	
**Maternal education**			
Not completed high school	12%	18%	χ^2^(4) = 7.59, *p* = 0.108
Completed high school	34%	33%	
Completed university	26%	23%	
Completed postgradual education	4%	1%	
Missing	25%	25%	
**Materinal smoking**			
Not smoking during pregnancy	66%	67%	χ^2^(1) = 0.10, *p* = 0.748
Smoking during pregnancy	9%	10%	
Missing	25%	22%	

**TABLE 2:** Biological aging and its predictors–descriptive statistics.

	Descriptive Statistics					Women					Men				
Measure	*N*	Min	Max	Mean	SD	*N*	Min	Max	Mean	SD	*N*	Min	Max	Mean	SD
**Biological aging**															
BioAGE	260	−2.84	4.39	0.00	1.19	125	−2.84	4.20	−0.03	1.27	135	−2.36	4.39	0.03	1.12
Current biological age	260	26.07	34.20	28.98	1.37	125	26.07	34.20	28.95	1.42	135	26.17	33.39	29.00	1.33
**BMI and body fat in late 20s**															
BMI	262	16.3	40.3	24.26	4.03	126	16.3	40.3	23.54	4.15	136	16.8	38.8	24.93	3.80
Overall body fat	262	5.0	47.4	23.04	9.07	126	7.9	47.4	29.35	7.34	136	5.0	35.2	17.20	6.14
Subcutaneous fat	262	3.9	33.8	13.14	5.95	126	6.1	33.8	14.72	6.07	136	3.9	33.1	11.68	5.46
Visceral fat	262	1.0	15.5	3.86	2.62	126	1.0	11.5	3.13	2.11	136	1.0	15.5	4.53	2.87
**BMI and body fat in early 20s**															
BMI	110	15.1	37.2	23.12	3.39	54	15.1	28.0	22.01	2.81	56	18.5	37.2	24.20	3.56
Overall body fat	109	14.7	45.0	28.30	6.49	54	14.7	45.0	29.79	6.62	55	17.4	40.0	26.84	6.06
Subcutaneous fat	110	5.5	28.5	13.57	5.33	47	6.3	28.5	14.25	4.91	63	5.5	27.8	13.07	5.61
**Change in BMI and body fat (early–late 20s)**															
BMI	110	−10.8	8.2	0.84	2.41	54	−4.8	8.2	0.77	2.24	56	−10.8	7.7	0.90	2.58
Overall body fat	110	−55.70	22.70	−5.93	8.59	54	−18.70	22.70	−1.66	6.30	56	−55.70	1.40	−10.06	8.53
Subcutaneous fat	110	−18.13	17.13	−1.38	5.62	47	−15.63	13.50	−1.04	5.28	63	−18.13	17.13	−1.63	5.90
**Puberty development**															
First period (years)	117	10	15	12.79	1.12	117	10	15	12.79	1.12					
Women: breast	62	1	4	2.10	0.84	62	1	4	2.10	0.84					
Women: pubic hair	62	1	4	1.94	1.02	62	1	4	1.94	1.02					
Men: genital	71	1	3	1.77	0.68						71	1	3	1.77	0.68
Men: pubic hair	69	1	3	1.39	0.57						69	1	3	1.39	0.57
**Birth outcomes**															
Birth weight (g)	256	1780	4600	3316	502	123	1780	4600	3159	476	133	1850	4600	3461	483
Bright length (cm)	256	40.0	56.0	50.25	2.36	123	40.0	54.0	49.46	2.45	133	43.0	56.0	50.98	2.01
Gestation (weeks)	133	37.43	42.43	39.97	1.12	66	37.43	42.00	39.88	1.08	67	37.43	42.43	40.05	1.17

### 2.2. Procedures

#### 2.2.1. Anthropometric measures

Weight and height were measured once at birth and twice in young adulthood (age 23–24, age 28–30). BMI in young adulthood was calculated as the ratio of the participant’s weight (kg) and height (m^2^). Total body fat and amount of visceral fat in young adulthood were estimated by bio-impedance using the scale Tanita BC-545 N. The bio-impedance scale was used in a standardized manner for all participants; the procedure followed the collection of fasting blood sample, and all participants were provided water during the consent procedure. All participants were also instructed not to drink alcohol the day before. Subcutaneous fat in young adulthood was measured with skinfold calipers at four locations (biceps, triceps, suprailia, and under scapula) using a standard procedure and the mean of these four measures (in millimeters) was used in the subsequent analyses.

#### 2.2.2. Gestational age

Gestational age was calculated as the difference between the date of birth and the ultrasound-based date of conception.

#### 2.2.3. Puberty development and onset of puberty

At the age of 11, pediatricians assessed the development of secondary sexual characteristics (breasts in women, penis in men, and pubic hair in both sexes) on a scale from 1 (least developed) to 4 (most developed). Participants with less developed secondary sexual characteristics at the age of 11 were classified as the early puberty onset group. In women, the age of menarche served as an additional predictor of puberty onset.

#### 2.2.4. Biomarkers in young adulthood

In the late 20s, forced expiratory volume in one second (FEV1) was calculated using MIR Smart One Spirometer. Systolic and diastolic blood pressure were assessed according to standard protocols. Blood samples were taken in the morning before the first meal. Cholesterol, C-reactive protein (CRP), glucose, albumin, creatinine, urea nitrogen serum levels (mg/dL) as well as alkaline phosphatase activity in serum (U/L) were measured on ROCHE analyzer (Cobas Integra 400, Roche diagnostics). The percentage of glycated hemoglobin was calculated based on glucose levels according to published equations and recommendations of the international consensus statement (34–37).

#### 2.2.5. Calculation of biological age and BioAGE in young adulthood

Biological age was calculated using Klemera-Doubal Method (KDM), available through the R package “Bio-Age” (9) that applies a 9-biomarker algorithm including forced expiratory volume in one second (FEV1), blood pressure (systolic), glycated hemoglobin, total cholesterol, C-reactive protein, creatinine, urea nitrogen, albumin, and alkaline phosphatase (see Supplementary Table 1 for descriptive statistics of biomarkers). The difference between biological age and chronological age (BioAGE) thus reflects accelerated/decelerated aging.

### 2.3. Statistical analysis

All statistical analyses were performed in SPSS version 28.0.0 (IBM SPSS Statistics). First, we assessed the distribution of data, and variables that did not follow a normal distribution were transformed using logarithmic transformation. Outliers that were greater than three standard deviations were removed from the analysis.

Measures of secondary sexual characteristics were fed into Two-Step Cluster Analysis (separate for both sexes) using Schwarz’s Bayesian Criterion to automatically detect clusters. Linear regression was used to assess the predictors of BioAGE. In each model where men and women were treated as one group, sex and the interaction between sex and the predictor were treated as covariates. The significant predictors were then used in a hierarchical multiple linear regression to assess the multiple predictors of BioAGE within a single model. Predictors entered the model following the order of the lifetime: 1. Birth length, 2. Puberty onset, 3. Fat measures in adulthood (visceral and subcutaneous simultaneously). Two analogous models were estimated: one for the whole group with sex as a covariate, and another one for women only, where the year of the first menarche was used as a more precise measure of puberty onset. Simple group differences were analyzed using an independent samples *t*-test. Group by puberty onset interaction was assessed using two-way ANOVA. Multiple comparisons were corrected using the False Discovery Rate (FDR) method and thus FDR-corrected *p*-values larger than 0.05 were considered significant.

## 3. Results

### 3.1. Biological aging in late 20s

**FIGURE 1:**
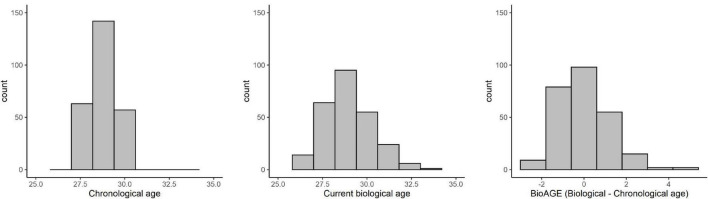
Distribution of chronological age, current biological age, and BioAGE.

While all participants were 28–30 years old, their current biological age ranged from 26.07 to 34.20 years, and thus their BioAGE ranged from −2.84 to 4.39 years (Figure 1).

### 3.2. Does BMI and body fat in the late 20s predict biological aging in the late 20s?

**FIGURE 2:**
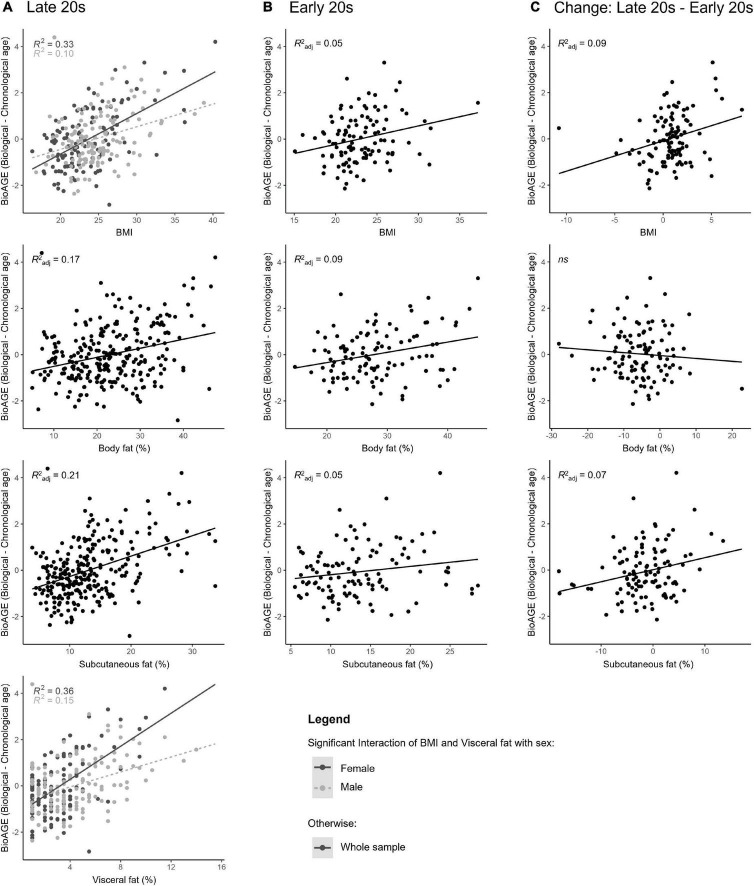
BMI and body fat in young adulthood as predictors of biological aging. Accelerated biological aging in the late 20s was predicted not only by BMI and body fat measured in the late 20s **(A)**, but also by BMI and body fat in the early 20s **(B)**, as well as the change in BMI and body fat over the 5-year period between the measurements **(C)**.

BioAGE in the late 20s (Figure 2A) was predicted by higher BMI [R^2^_*adj*_ = 0.22, F(3,256) = 25.03, β = 0.10, *p* < 0.001], overall body fat [R^2^_*adj*_ = 0.17, F(3,256) = 19.22, β = 0.07, *p* < 0.001], subcutaneous fat [R^2^_*adj*_ = 0.21, F(3,256) = 23.35, β = 0.08, *p* < 0.001] and visceral fat [R^2^_*adj*_ = 0.25, F(3,256) = 30.44, β = 0.16, *p* < 0.001]. In addition, we found an interaction effect between sex and BMI (for every unit of BMI increase, BioAGE in women increased 0.08 years more than in men, β = 0.79, *p* = 0.022) and between sex and visceral fat (for every percent increase in visceral fat, BioAGE in women increased 0.2 years more than in men, β = 0.35, *p* < 0.001).

*Post-hoc* regressions in each sex revealed that BioAGE in late 20s was predicted by BMI in both women [R^2^ = 0.33, F(1,123) = 61.20, β = 0.58, *p* < 0.001] and men [R^2^ = 0.10, F(1,133) = 15.13, β = 0.32, *p* < 0.001], and by visceral fat in both women [R^2^ = 0.35, F(1,123) = 68.09, β = 0.60, *p* < 0.001] and men [R^2^ = 0.15, F(1,133) = 23.92, β = 0.39, *p* < 0.001].

### 3.3. Does BMI and body fat in the early 20s predict biological aging in the late 20s?

BioAGE in the early 20s (Figure 2B) was significantly associated with higher BMI [R^2^_*adj*_ = 0.05, F(3,105) = 3.09, β = 0.04, *p* = 0.037], overall body fat [R^2^_*adj*_ = 0.09, F(3,104) = 4.54, β = 0.04, *p* = 0.009] and subcutaneous fat [R^2^_*adj*_ = 0.05, F(3,105) = 2.78, β = 0.02, *p* = 0.049]. There were no interactions with sex (*p* = 0.022).

### 3.4. Does the change in BMI and body fat from the early to late 20s predict biological aging in the late 20s?

Decrease of BMI [R^2^_*adj*_ = 0.09, F(3,105) = 4.39, β = 0.08, *p* = 0.009] and subcutaneous fat [R^2^_*adj*_ = 0.07, F(3,105) = 3.77, β = 0.04, *p* = 0.018], but not of overall body fat (*p* = 0.431) over the 5-year period in young adulthood were associated with lower BioAGE in late 20s (Figure 2C). There were no interactions with sex (*p* = 0.189).

### 3.5. Does the timing of puberty onset predict biological aging in the late 20s?

The puberty data were classified into two categories based on the development of secondary sexual characteristics at the age of 11 in both women (late onset of puberty: *n* = 17, early onset of puberty, *n* = 45) and men (late onset of puberty: *n* = 26, early onset of puberty: *n* = 43).

**FIGURE 3:**
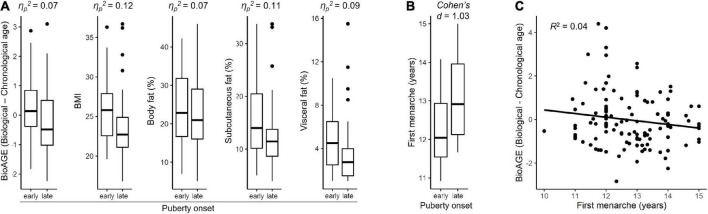
Pubertal timing. Differences between early and late onset of puberty based on secondary sexual characteristics in relation to accelerated biological aging, BMI, and fat measures in late 20s within whole sample **(A)** and in relation to age of first menarche in women **(B)**. Accelerated biological aging (BioAGE) in women predicted by earlier age of first menarche **(C)**.

Two-way ANOVA revealed a significant effect of puberty timing on BioAGE, BMI as well as body fat. Early puberty onset group had more accelerated BioAGE [η_*p*_^2^ = 0.07, F(1,125) = 10.01, *p* = 0.004], higher BMI [η_*p*_^2^ = 0.12, F(1,127) = 17.90, *p* < 0.001], overall body fat [η_*p*_^2^ = 0.07, F(1,127) = 10.05, *p* = 0.004], subcutaneous fat [η_*p*_^2^ = 0.12, F(1,127) = 17.99, *p* < 0.001] as well as visceral fat [η_*p*_^2^ = 0.09, F(1,127) = 12.11, *p* = 0.003] in late 20s than the late puberty onset group (Figure 3A). There was no significant interaction between puberty timing and sex on any of the dependent variables (*p* = 0.053).

Sex-specific *post-hoc* analyses showed that the effects of puberty onset were driven by women (Supplementary Figure 2). In women, early puberty onset group had more accelerated BioAGE [η_*p*_^2^ = 0.11, F(1,125) = 16.06, *p* = 0.001], higher BMI [η_*p*_^2^ = 0.13, F(1,127) = 18.62, *p* = 0.001], higher overall body fat [η_*p*_^2^ = 0.07, F(1,127) = 9.85, *p* = 0.009], subcutaneous fat [η_*p*_^2^ = 0.11, F(1,127) = 16.33, *p* = 0.001] and visceral fat [η_*p*_^2^ = 0.08, F(1,127) = 10.83, *p* = 0.007] than late puberty onset group (Supplementary Figure 2). No similar effects of puberty onset were found in men (*p* = 0.261). Complete statistics is reported in Supplementary Table 3.

Women with earlier onset of puberty experienced earlier first menarche [Cohen’s d = 1.03, t(56) = 3.93, *p* < 0.001] (Figure 3B) and earlier first menarche predicted higher BioAGE [R^2^ = 0.04, F(1,114) = 6.13, β = 0.26, *p* = 0.015] (Figure 3C).

### 3.6. Does birth weight, length, or gestational age predict accelerated biological aging in the late 20s?

**FIGURE 4:**
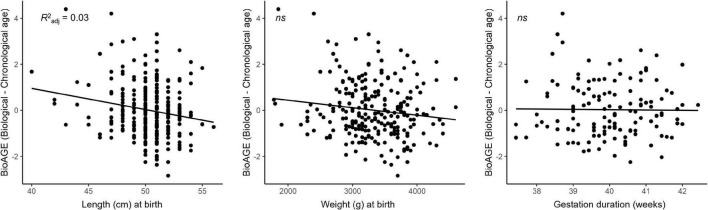
Accelerated biological aging in the late 20s was predicted by birth length but not birth weight or duration of gestation.

Shorter birth length was associated with higher BioAGE [R^2^_adj_=0.03, F(3,250)=3.61, β=−0.08, *p* = 0.042], but no significant relationship emerged between birth weight (*p* = 0.127) or the duration of gestation (*p* = 0.843) and BioAGE (see Figure 4). There were no interactions with sex (*p* = 0.382).

### 3.7. Single model of accelerated biological aging combining predictors from birth to adulthood

Multiple regression evaluating the effects of birth length, puberty onset, and visceral and subcutaneous fat in the late 20s on BioAGE in the whole sample showed that all the predictors together explained 21% of the variance in biological aging [R^2^_*adj*_ = 0.21, F(5,119) = 7.59, *p* > 0.001]. While birth length explained 3.7% of the variance [F(122,1) = 6.49, *p* = 0.012], early puberty onset explained additional 5.2% [F(121,1) = 8.00, *p* = 0.005], and visceral fat another 12.1% [F(119,2) = 10.28, *p* < 0.001]. For every cm of birth length, BioAGE decreased by 0.119 years (β = −0.236, *p* = 0.012). Participants with early puberty onset had on average 0.56 years more advanced BioAGE than those with late puberty onset (β = −0.244, *p* = 0.005). For every% of visceral fat, BioAGE increased by 0.167 years (β = 0.361, *p* = 0.006). The effect of subcutaneous fat did not reach significance in the multiple regression (*p* = 0.737). Complete statistics with all regressors are in Supplementary Table 4A.

Similar multiple regression in women, where we could use the age of menarche as a more accurate measure of puberty timing, showed that the whole model explained even 30% of the variance [R^2^_*adj*_ = 0.30, F(4,108) = 13.01, *p* > 0.001]. While birth length explained 6.9% of the variance [F(111,1) = 9.26, *p* = 0.003], adding year of first menarche explained additional 0.9% although not significant [F(110,1) = 2.11, *p* = 0.149], and body fat another 22.2% [F(1008,2) = 18.47, *p* < 0.001]. For every cm of birth length, BioAGE decreased by 0.137 years (β = −0.28, *p* < 0.003). For every% of visceral fat, BioAGE increased by 0.247 years (β = 0.445, *p* < 0.001). The effect of first menarche (*p* = 0.149) and subcutaneous fat (*p* = 0.634) did not reach significance in the multiple regression. Complete statistics with all regressors are in Supplementary Table 4B.

## 4. Discussion

We studied biological aging in young adults from the ELSPAC-CZ prenatal birth cohort and demonstrated that accelerated biological aging in young adulthood was associated with higher BMI as well as higher overall, subcutaneous, and visceral body fat, with visceral fat showing the strongest association. Moreover, we showed that the effects of BMI and body fat on biological aging are stable–present in both early and late 20s–and reach a greater effect size in women as compared to men. Most importantly, we demonstrated that reduction of BMI over the 5-year period between the measurements was associated with decelerated biological aging, suggesting that reducing weight over a relatively short period of time during adulthood can possibly slow down the pace of biological aging.

These findings extend previous prospective cohort studies, which linked higher BMI (10–12) and body fat (38) with increased mortality. They also support research by others reporting associations between higher BMI and accelerated epigenetic aging (39–43). While the mechanisms explaining the relationships between higher BMI and accelerated epigenetic aging remain to be clarified, the associations suggest the existence of a shared developmental mechanism (16).

The relationships between accelerated biological aging and higher BMI in both the late and early 20s demonstrate the stability of the effect. But interestingly, a reduction of BMI over the 5-year period predicted lower BioAGE, suggesting that we might be able to slow down the speed of our biological aging by relatively accessible management options. This is in agreement with previous research suggesting dieting (44) and caloric restriction (15) as means to increase lifespan. Consistently, exercise was found to affect epigenetic changes in DNA methylation (45), histone modification (46), chromatin modifications (47), and non-coding RNAs (48) that are associated with aging (16). Further research is needed to assess the link between exercise and BioAGE.

Early puberty onset was another key predictor of accelerated biological aging, particularly in women. This is in agreement with previous studies that found a relationship between earlier menarche and accelerated epigenetic aging in women (5, 23). While Binder et al. (23) reported a relationship between epigenetic aging and menarche but not breast development, we found the effects of both menarche and breast development (together with pubic hair development). These divergent findings might be attributed to differences in methodology: first, our measure of aging is composed of wider selection of biomarkers; second, compared to the onset of breast development used by Binder et al. (23), we measured the degree of development at the age of 11.

We found only limited evidence for the hypothesized early life origins of biological aging. In particular, newborns who were shorter (but not lighter or younger) at birth were aging faster in their late 20s. This might be related to the fact that all our participants fell within the healthy range and the low variance in gestational age and birth weight might not have allowed us to detect any significant relationships with biological aging in young adulthood.

Finally, combining predictors of BioAGE from birth to adulthood allowed us to explain up to 21% of the variance in the whole sample and up to 30% of the variance in the women’ group. Interestingly, visceral but not subcutaneous fat was a significant predictor of BioAGE. While both types of fat have been associated with increased morbidity (49, 50), there are indications that visceral fat is a more relevant predictor of cardiometabolic diseases than subcutaneous fat (51–53). Our findings suggest that higher levels of visceral fat might have important health consequences not only for the risk of cardiometabolic diseases but also aging and that high levels of visceral fat have particular negative impact on women. The puberty timing measured by secondary sexual characteristics was another significant predictor of accelerated aging in both the whole sample as well as women only. However, the timing of puberty measured by the first menarche did not constitute a significant predictor of BioAGE in women, when birth length and measures of visceral and subcutaneous fat in adulthood were accounted for. It must be noted that the effect of the first menarche was rather small even when considered alone and its lack of effect in the multiple regression model might be attributed to the limited sample size.

Overall, our findings support the Life History Theory, according to which early pubertal maturation can be accounted for accelerated biological aging (21). The rationale behind the theory is that the adaptation of an organism to early live adversity is reflected in accelerated aging. This leads to earlier pubertal maturation which increases the organism’s chance of reproduction before dying. However, the payoff for the earlier pubertal maturation is decreased health in adulthood which is associated with aging, leading to increased morbidity and premature mortality.

Our study has several limitations that need to be acknowledged. First, the sample size is considerably small, which can be, at least in part, attributed to the longitudinal design of our study. Second, members of our prenatal birth cohort were not born preterm and had a healthy birth weight, limiting the possibility to study the impact of birth outcomes. Third, while our study uses longitudinal data for the predictors, the blood sample to estimate biological age was collected only at a single point at the late 20s. Further research is needed to assess the stability of the biological age gap (BioAGE) across the lifespan. Fourth, potential confounders such as lifestyle and dietary behavior might have affected the results and should be considered by future studies. Finally, this is a correlational study and therefore does not allow us to prove causal relationships between BioAGE and its predictors.

In conclusion, using longitudinal data on the ELSPAC-CZ prenatal birth cohort, we demonstrated that birth length, puberty timing, and visceral fat predict biological aging in young adulthood. In particular, the results of our study provide comprehensive support for the Life History Theory, suggesting that early life adversity might trigger accelerated aging, which in turn leads to earlier pubertal timing but decreasing fitness in adulthood, reflected by higher visceral fat and BMI. Moreover, we discovered that a reduction of BMI in young adulthood might slow down biological aging.

## Data availability statement

The raw data supporting the conclusions of this article will be made available by the authors, without undue reservation.

## Ethics statement

The studies involving human participants were reviewed and approved by European Longitudinal Study of Pregnancy and Childhood (ELSPAC-CZ) Ethics Committee. The patients/participants provided their written informed consent to participate in this study.

## Author contributions

MJ: investigation, formal analysis, and writing the original draft. LZ: investigation. PP: resources. LA: resources. MB: review and editing. KM: conceptualization, methodology, supervision, funding acquisition, review, and editing. All authors contributed to the article and approved the submitted version.
