# Supplementary material for: Birth outcomes, puberty onset, and obesity as long-term predictors of biological aging in young adulthood
Source: Front Nutr. 2023 Jan 10;9:1100237. doi: 10.3389/fnut.2022.1100237 (PMC9873383; doi:10.3389/fnut.2022.1100237)
Supplement: Supplementary file 1 [file Data_Sheet_1.PDF]

frontiers

Supplementary Material

Supplementary table S1. Descriptive statistics of biomarkers used to estimate biological age.

Biomarker	Total (N = 260)		Female (N = 125)		Male (N = 135)	
	Mean	SD	Mean	SD	Mean	SD
Blood pressure (systolic)	121.72	12.95	115.65	10.59	127.34	12.42
Forced expiratory volume in one second (FEV1)	4165.04	929.50	3490.80	609.26	4789.33	713.92
Albumin	4.71	0.22	4.62	0.22	4.79	0.18
Alkaline phosphatase	66.18	18.00	64.98	19.87	67.29	16.07
Total cholesterol	166.27	30.13	167.75	30.34	164.90	29.99
Creatinine	0.85	0.15	0.74	0.10	0.94	0.11
C-reactive protein	0.18	0.27	0.24	0.35	0.13	0.14
Glycated hemoglobin	4.80	0.31	4.75	0.28	4.85	0.32
Urea nitrogen	0.02	0.01	0.02	0.01	0.03	0.01

Supplementary figure S1. Sample selection flowchart

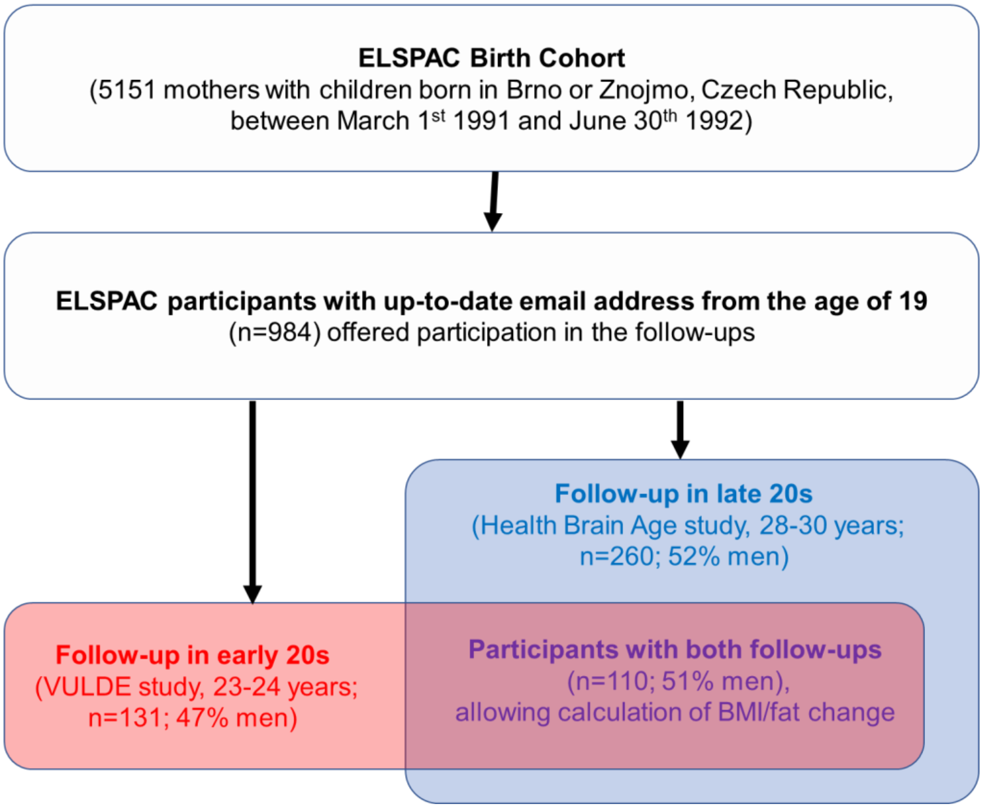

Supplementary table S2. Predictors of Accelerated Biological Aging.

				model					Unstandardized Coefficients		Standardized Coefficients		
Predictor	R	R^2_{adj}	SE	F	df1	df2	p	Coefficients	B	SE	Beta	t	p
**BMI and body fat in late 20s**													
BMI	0.48	0.22	1.06	25.03	3	256	<0.001	(Constant)	-2.40	0.62		-3.86	<0.001
								predictor	0.10	0.02	0.32	3.94	<0.001
								sex	-1.75	0.83	-0.74	-2.12	0.035
								predictor*sex	0.08	0.03	0.79	2.30	0.022
overall fat (%)	0.43	0.17	1.08	19.22	3	256	<0.001	(Constant)	-1.11	0.28		-3.91	<0.001
								predictor	0.07	0.02	0.51	4.24	<0.001
								sex	-1.36	0.49	-0.57	-2.76	0.006
								predictor*sex	0.02	0.02	0.21	0.80	0.425
subcutaneous fat (%)	0.46	0.21	1.06	23.35	3	256	<0.001	(Constant)	-0.85	0.22		-3.78	<0.001
								predictor	0.08	0.02	0.37	4.27	<0.001
								sex	-0.84	0.34	-0.35	-2.50	0.013
								predictor*sex	0.04	0.02	0.26	1.54	0.124
visceral fat (%)	0.51	0.25	1.03	30.44	3	256	<0.001	(Constant)	-0.69	0.17		-4.06	<0.001
								predictor	0.16	0.03	0.34	4.93	<0.001
								sex	-0.45	0.24	-0.19	-1.91	0.057
								predictor*sex	0.20	0.05	0.35	3.58	<0.001
**BMI and body fat in early 20s**													
BMI	0.29	0.05	1.02	3.09	3	105	0.030	(Constant)	-0.93	0.96		-0.97	0.333
								predictor	0.04	0.04	0.14	1.12	0.265
								sex	-1.98	1.46	-0.95	-1.36	0.177
								predictor*sex	0.08	0.06	0.90	1.32	0.189
overall fat (%)	0.34	0.09	1.00	4.54	3	104	0.005	(Constant)	-0.90	0.62		-1.44	0.153
								predictor	0.04	0.02	0.24	1.72	0.089
								sex	-1.06	0.89	-0.51	-1.19	0.235
								predictor*sex	0.02	0.03	0.35	0.75	0.453
subcutaneous fat (%)	0.27	0.05	1.07	2.78	3	105	0.045	(Constant)	-0.20	0.34		-0.58	0.563
								predictor	0.02	0.02	0.10	0.86	0.391
								sex	-1.09	0.59	-0.50	-1.84	0.068
								predictor*sex	0.05	0.04	0.37	1.30	0.196
**Change in BMI and body fat between late vs. early 20s**													
BMI	0.33	0.09	1.00	4.39	3	105	0.006	(Constant)	0.07	0.14		0.48	0.633
								predictor	0.08	0.06	0.18	1.47	0.144
								sex	-0.32	0.20	-0.15	-1.56	0.122
								predictor*sex	0.11	0.08	0.17	1.29	0.200
overall fat (%)	0.16	0.00	1.05	0.93	3	104	0.431	(Constant)	0.30	0.28		1.08	0.282
								predictor	0.02	0.03	0.11	0.64	0.524
								sex	-0.44	0.31	-0.21	-1.39	0.167
								predictor*sex	-0.04	0.03	-0.16	-1.09	0.277
subcutaneous fat (%)	0.31	0.07	1.05	3.77	3	105	0.013	(Constant)	0.16	0.14		1.11	0.272
								predictor	0.04	0.02	0.22	1.77	0.079
								sex	-0.33	0.21	-0.15	-1.57	0.119
								predictor*sex	0.03	0.04	0.08	0.68	0.498
**Birth outcomes**													
birth weight (in g)	0.16	0.01	1.19	2.24	3	250	0.085	(Constant)	0.89	0.75		1.19	0.236
								predictor	<0.01	<0.01	-0.10	-1.15	0.250
								sex	0.74	1.04	0.31	0.71	0.479
								predictor*sex	<0.01	<0.01	-0.37	-0.88	0.382
birth length (in cm)	0.20	0.03	1.18	3.61	3	250	0.014	(Constant)	4.23	2.60		1.62	0.105
								predictor	-0.08	0.05	-0.16	-1.61	0.108
								sex	1.88	3.38	0.79	0.56	0.579
								predictor*sex	-0.04	0.07	-0.86	-0.62	0.536
gestation length (in weeks)	0.08	-0.02	1.22	0.28	3	128	0.843	(Constant)	-1.59	5.16		-0.31	0.759
								predictor	0.04	0.13	0.04	0.30	0.766
								sex	4.26	7.63	1.77	0.56	0.577
								predictor*sex	-0.10	0.19	-1.70	-0.54	0.591

Supplementary table S3. Two-way ANCOVA differences between early and late onset of puberty with interaction by sex in biological aging, BMI, and measures of body fat.

effect / variable	η_p^2	Type III Sum of Squares	df1	df2	Mean Square	F	p	Late Mean	Late SE	Early Mean	Early SE
**puberty onset**											
BioAGE	0.07	10.59	1	125	10.59	10.01	0.004	-0.26	0.11	0.35	0.16
BMI	0.12	231.22	1	127	231.22	17.90	<0.001	23.15	0.38	26.03	0.56
overall body fat	0.07	377.61	1	127	377.61	10.05	0.004	21.83	0.65	25.50	0.96
subcutaneous fat	0.12	563.16	1	127	563.16	17.99	<0.001	11.86	0.60	16.34	0.87
visceral fat	0.09	68.06	1	127	68.06	12.11	0.003	3.22	0.25	4.78	0.37
**interaction of puberty onset * sex**											
BioAGE	0.06	8.72	1	125	8.72	8.24	0.053				
BMI	0.04	66.64	1	127	66.64	5.16	0.136				
overall body fat	0.02	91.15	1	127	91.15	2.43	0.312				
subcutaneous fat	0.03	106.91	1	127	106.91	3.42	0.245				
visceral fat	0.02	12.29	1	127	12.29	2.19	0.312				
**posthoc: puberty onset in women**											
BioAGE	0.11	16.99	1	125	16.99	16.06	0.001	-0.46	0.16	0.71	0.25
BMI	0.13	240.50	1	127	240.50	18.62	0.001	22.17	0.54	26.59	0.87
overall body fat	0.07	369.83	1	127	369.83	9.85	0.009	27.73	0.91	33.21	1.49
subcutaneous fat	0.11	511.19	1	127	511.19	16.33	0.001	13.12	0.83	19.56	1.36
visceral fat	0.08	60.86	1	127	60.86	10.83	0.007	2.46	0.35	4.68	0.57
**posthoc: puberty onset in men**											
BioAGE	<0.01	0.05	1	125	0.05	0.05	0.903	-0.06	0.16	-0.01	0.20
BMI	0.02	28.68	1	127	28.68	2.22	0.339	24.13	0.55	25.47	0.70
overall body fat	0.01	56.51	1	127	56.51	1.50	0.445	15.92	0.93	17.79	1.20
subcutaneous fat	0.03	103.70	1	127	103.70	3.31	0.261	10.59	0.85	13.12	1.10
visceral fat	0.02	13.02	1	127	13.02	2.32	0.339	3.99	0.36	4.88	0.46

Supplementary figure S2. Differences between early/late puberty timing effects – breakdown by sex

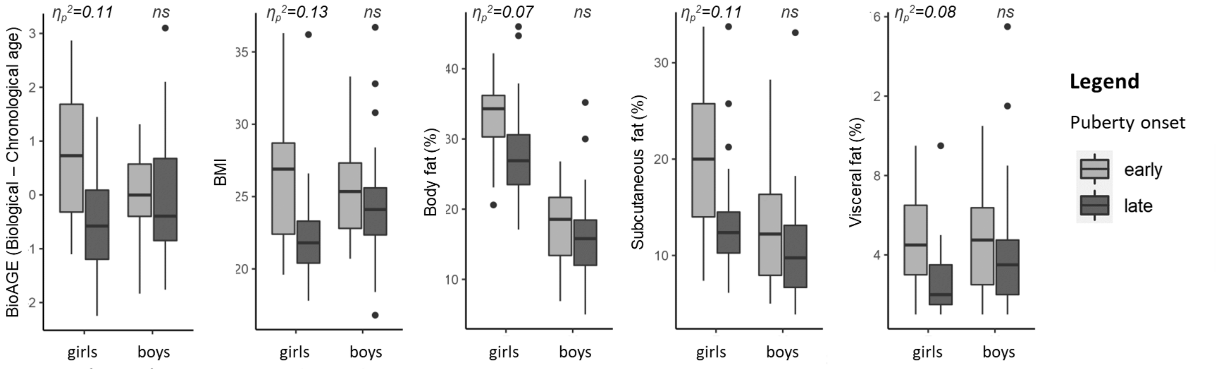

Supplementary table S4A. Hierarchical model of biological aging with birth length, puberty timing and body fat as predictors within whole sample.

Predictors added at each step	R	R^2_{adj}	SE	model F	model df1	model df2	model p	Coefficients	Unstandardized Coefficients B	Unstandardized Coefficients SE	Standardized Coefficients Beta	t	p
1. sex (control variable)	0.04	-0.01	1.08	0.21	1	123	0.651	(Constant)	-0.01	0.13		-0.10	0.924
								sex	-0.09	0.19	-0.04	-0.45	0.651
2. birth length (cm)	0.23	0.04	1.06	3.35	2	122	0.038	(Constant)	6.03	2.38		2.54	0.012
								sex	-0.25	0.20	-0.11	-1.24	0.218
								birth length (cm)	-0.12	0.05	-0.24	-2.55	0.012
3. puberty timing (early=0, late=1)	0.33	0.09	1.03	5.03	3	121	0.003	(Constant)	6.03	2.31		2.61	0.010
								sex	-0.20	0.19	-0.09	-1.00	0.318
								birth length (cm)	-0.11	0.05	-0.22	-2.46	0.015
								late puberty onset (=1)	-0.56	0.20	-0.24	-2.83	0.005
4. subcutaneous fat (%), visceral fat (%)	0.49	0.21	0.96	7.59	5	119	<0.001	(Constant)	4.39	2.25		1.95	0.053
								sex	-0.06	0.23	-0.03	-0.27	0.788
								birth length (cm)	-0.10	0.04	-0.20	-2.29	0.024
								late puberty onset (=1)	-0.25	0.20	-0.11	-1.24	0.216
								subcutaneous fat (%)	0.01	0.02	0.05	0.34	0.737
								visceral fat (%)	0.17	0.06	0.36	2.78	0.006

Supplementary table S4B. Hierarchical model of biological aging with birth length, puberty timing and body fat as predictors for women subsample.

Predictors added at each step	R	R^2_{adj}	SE	model F	model df1	model df2	model p	Coefficients	Unstandardized Coefficients B	Unstandardized Coefficients SE	Standardized Coefficients Beta	t	p
1. birth length (cm)	0.28	0.07	1.23	9.26	1	111	0.003	(Constant)	6.75	2.22		3.04	0.003
								birth length (cm)	-0.14	0.04	-0.28	-3.04	0.003
2. first menarche (in years)	0.31	0.08	1.22	5.73	2	110	0.004	(Constant)	8.41	2.49		3.38	0.001
								birth length (cm)	-0.13	0.04	-0.27	-2.93	0.004
								first menarche (in years)	-0.15	0.10	-0.13	-1.45	0.149
3. subcutaneous fat (%), visceral fat (%)	0.57	0.30	1.06	13.01	4	108	<0.001	(Constant)	6.91	2.21		3.12	0.002
								birth length (cm)	-0.14	0.04	-0.29	-3.60	<0.001
								first menarche (in years)	-0.07	0.09	-0.06	-0.74	0.459
								subcutaneous fat (%)	0.01	0.02	0.05	0.48	0.634
								visceral fat (%)	0.25	0.06	0.45	3.84	<0.001
